# The epidemiology of anal incontinence and symptom severity scoring

**DOI:** 10.1093/gastro/gou005

**Published:** 2014-02-27

**Authors:** Avinoam Nevler

**Affiliations:** Department of Surgery and Transplantation, the Dr. Pinchas Borenstein Talpiot Medical Leadership Program 2012, Chaim Sheba Medical Center, Tel Aviv University, Ramat Gan, Israel (Affiliated to Sackler Medicine School)

**Keywords:** anal incontinence, epidemiology, symptom severity scoring

## Abstract

For many patients, anal incontinence (AI) is a devastating condition that can lead to social isolation and loss of independence, contributing to a substantial economic health burden, not only for the individual but also for the allocation of healthcare resources. Its prevalence is underestimated because of poor patient reporting, with many unrecorded but symptomatic cases residing in nursing homes. Endosonography has improved our understanding of the incidence of post-obstetric sphincter tears that are potentially suitable for repair and those cases resulting from anorectal surgery, most notably after fistula and hemorrhoid operations. The clinical scoring systems assessing the severity of AI are discussed in this review, along with their limitations. Improvements in the standardization of these scales will advance our understanding of treatment response in an era where the therapeutic options have multiplied and will permit a better comparison between specific therapies.

## PREVALENCE OF ANAL INCONTINENCE

Anal incontinence (AI) is a chronic, debilitating condition resulting from an inability to effectively restrict the passage of fecal material through the anal canal. This leads to loss of control over bowel movements and, in severe cases, involuntary defecation. AI leads to severe impairment of the quality of life, frequent infectious medical complications, numerous absences from school and work and overwhelming psychological stress to the patient and his or her family. The reported incidence of AI varies worldwide. During the past two decades, most studies have reported a prevalence of 2.2—7% in the general population [1, 2]; however, in recent years and with a growing public awareness of the condition, there has been a rise in the reported prevalence of AI up to 8.3% amongst non-institutionalized adults [3]. This condition has an even higher prevalence in the elderly, at 6–19% [4–6], and particularly amongst residents of nursing homes, where it has been recorded in nearly 50% of cases questioned [7, 8].

AI has an equal prevalence in males and females [2, 9–11]; however, it has been shown to be more prevalent in multiparous women, those women who have undergone instrument-assisted vaginal deliveries and in women following traumatic deliveries involving significant vaginal tears [12–14]. The likelihood is that these estimates are under-represented as part of dual, anal-plus-urinary incontinence. In this regard, a population-based study from Minnesota, surveying 1500 people over the age of 50 years, showed that 5.4% of men and 9.4% of women, reported combined incontinence [15]. In those with AI, the prevalence of urinary incontinence was 51.1% in men and 59.6% in women with stress type incontinence in 42%, detrusor instability in 11%, mixed urinary incontinence in 37% and no detectable urodynamic anomaly found in 6% of cases [16]. Conversely, in those with primary urinary incontinence and/or pelvic organ prolapse, Meschia *et al.* noted that 20% also suffered from AI [17]. These findings are similar to those reported by the Cleveland Clinic Florida, where the incidence of urinary incontinence in those surgically treated for AI was 54%, rising to 64% in those requiring prolapse surgery [18]. In an interesting group of nulliparous Canadian teenage girls, 17% had occasional urinary incontinence, with 38% reporting minor gaseous AI. In this survey, 3% reported occasional major fecal soiling but the vast majority of the cohort did not believe that these symptoms were either abnormal or worth reporting to their medical practitioner [19]. Further, the overall incidence of AI is likely to be underestimated in nursing homes, where it is specifically associated with fecal impaction and disimpaction episodes, as well as in patients admitted with neurological disorders.

The loss of control over bowel movements has a major impact on social interactions and is associated with shame, embarrassment and social phobia. AI is therefore affected by low rates of self-reporting and therapy-seeking behavior [20]. In this respect, Aitola *et al.*, studying 162 subjects who reported episodes of bowel incontinence at least twice a month, showed that only 27% of cases had discussed the problem with their primary physician and only 10% had received treatment for the problem [21]. When questioned, 66% responded that they felt that they actually needed treatment, if it was available. These low rates of therapy-seeking behavior persist in the post-partum group, with only 10–16% of women seeking medical help at between 6 months and 2 years after delivery if there are persistent continence issues [22].

## ETIOLOGY OF ANAL INCONTINENCE

The causes of AI are diverse and shown in Table 1. The majority of severe AI develops because of a specific causative agent: most notably, obstetric trauma and anal surgery [23, 24]. Correlation of AI to different risk factors is somewhat limited, since the true incidence of minor AI is masked by the low reporting rate. Although specific obstetric risk factors for AI have been established, the literature on individual risk is still self-contradictory. In a study by de Parades *et al.*, the reported prevalence of AI in women following forceps delivery was 18.2% for flatus AI and 4.3% for fecal AI [14]. However, in a recent prospective study with a cohort of 449 vaginally parous participants, no significant difference was noted in AI prevalence between normal, vacuum or forceps delivery, or in those where there were vaginal tears [25]. Another important subgroup is the iatrogenic AI group. Iatrogenic AI is usually associated with anorectal, perineal or pelvic surgery or post-pelvic irradiation. Internal sphincterotomy and peri-anal fistulotomy/fistulectomy have been shown to be associated with a high incidence of fecal soiling (varying from 0–35%) [26]. Other perineal procedures, such as radical perineal and retropubic prostate surgery, result in an incidence of 7–18% of fecal soiling [27]. The impact of pelvic irradiation on the incidence and severity of AI is limited; however, it has been shown to be connected to fecal urgency, diarrhea and flatulence [28–31]. Neurological disorders comprise another unique sub-population of AI patients. The most common neurological cause for AI is a cerebrovascular accident (CVA) in which AI prevalence in the immediate and long-term periods is 30% and 15%, respectively. Other neurological causes include anoxic brain damage, cerebral palsy, spinal cord injury (SCI) and multiple sclerosis.

**Table 1.** Etiological factors contributing to anal incontinence

Trauma/obstetric	Neurological	Psychiatric	Inflammatory/infectious	Surgical	Congenital
Sphincter disruption	Cerebrovascular accident	Functional	Proctitis	Post-rectal resection	Anorectal malformations
Perineal tears	Spinal cord injury	Cognitive	Inflammatory bowel disease	Post-anorectal surgery	Spina bifida
Pudendal neuropathy	Multiple sclerosis		Severe perianal/ perineal sepsis	Anorectal trauma	Hirschprung's disease
	Peripheral neuropathy (e.g. diabetes mellitus)			Functional rectal disorders (e.g. rectocele)	
	Brain trauma, anoxic brain damage, cerebral palsy			Low anterior resection syndrome	

## SYMPTOM SEVERITY: GRADING SYSTEMS

When assessing the patient case history, direct questioning is required concerning the complaint, with details regarding prior anorectal and colorectal surgery. Despite the fact that specific questionnaires about the severity of AI and its impact on quality of life are in current worldwide use for many specialized conditions, (neurological disease and spinal cord injury or *cauda equina* syndrome in particular), there are presently no standardized sets of questions designed to elucidate the comprehensive set of factors involved in AI. Standardized assessment of symptoms of AI affords a better understanding of the effect of etiological causes (on a multifactorial background) as well as its functional impact on lifestyle and the directed use of specific treatment modalities. Most scoring systems incorporate similar parameters, which include the nature of the incontinence (flatus, liquid seepage, liquid incontinence, solid incontinence), the incontinence type (active awareness, passive non-awareness, urge incontinence), the quantity of loss, the frequency of incontinence episodes, and accompanying complaints such as abdominal/pelvic pain, obstructed defecation, urinary complaints (including incontinence and type), associated pelvic organ prolapse and, in some scoring instruments, a broad cognitive assessment.

Further standardization of clinical incontinence scales will afford better comparison of treatment modalities, both within and between treating departments. The simplest scale method was initially presented by Browning and Parks in 1983 [32]. This scale includes four main categories in which the lowest grade is normal continence and the highest is total incontinence: it was originally used to assess the success (or otherwise) of a posterior Parks' anal sphincter repair (Table 2).

**Table 2.** Browning and Parks' incontinence scale [32]

I	Normal continence (i.e. continent for solids, liquid stools and flatus)
II	Continent for solid and liquid stools but not for flatus
III	Continent for solid stools only. Usually presented with fecal leakage
IV	Complete incontinence

The Parks' scale, whilst easy to use and remember, had several major shortcomings as it did not address symptom severity, the frequency of incontinence episodes or any impact that the incontinence might have on health-related quality of life. As a result, paradoxically, in this scoring system, a patient presenting with daily leakage of large amounts of liquid stool would be scored less severely than one presenting with infrequent episodes of solid stool incontinence. Later scoring systems have added more relevant parameters pertaining to severity of incontinence and include the more commonly used Fecal Incontinence Quality of Life (Rockwood) Scale (FIQOL) [33], the Wexner Cleveland Clinic Florida Score [23], the St. Mark's (Vaizey) Score [34], and the Pescatori Score [35].

The Rockwood FIQOL score was introduced in 2000 and was used to evaluate psychometric health-related quality of life parameters in AI patients. The score is arranged on four psychosocial scales: lifestyle, coping/behavior, depression/self-perception, and embarrassment, and contains a total of 29 different items. The FIQOL was based on the sub-topics of the SF-36 general health survey, which have been proven to be reliable, comparable and reproducible in several studies and languages [36–38]. The scale does contain some redundant items that potentially could be removed without affecting overall validity and it has not been applied to patients with varying degrees of incontinence, nor has it been compared with anorectal manometry [39].

The Jorge-Wexner score is the most widely used instrument in assessing the efficacy of surgical therapies for AI, although it has yet to undergo formal validation studies during specific treatments. This scoring system cross-tabulates frequencies and different anal incontinence presentations (Gas/Liquid/Solid/Pad use/Need for lifestyle alterations) and sums the returned score to a total of 0–20 (where 0 = perfect continence and 20 = complete incontinence). Each of the incontinence presentations is graded equally in this scoring system and no psychometric items are included, other than the non-specific ‘Lifestyle Alterations' item (Table 3). The Wexner score does not take specific account of fecal urgency (*vide infra* St. Mark's scale), even in the absence of specific incontinence episodes, nor of the importance of the use of a pad in terms of continence, which are both given equal weighting. Pad use may also reflect urinary incontinence or patients' hygienic concerns, independently of episodes of incontinence; further, it does not assess the use of specific anti-diarrheal medications.

**Table 3.** The Jorge-Wexner incontinence score

Type of incontinence	Frequency				
	Never	Rarely	Sometimes	Usually	Always
Solid	0	1	2	3	4
Liquid	0	1	2	3	4
Gas	0	1	2	3	4
Wears pad	0	1	2	3	4
Lifestyle alteration	0	1	2	3	4

Never = 0; Rarely = <1/month; Sometimes = <1/week but >1/month; Usually = <1/day but >1/week; Always = >1/day.

The St. Mark's (Vaizey) score, published in 1999, is also commonly used in clinical studies and reports and was based on the Jorge-Wexner score but added two further items for assessment: the use of constipating medication and the presence of fecal urgency. The relative weighting of pad (or anal plug) use was decreased in this score, where the designers felt that such use may represent more the subjective fear of social embarrassment, rather than actual frequency. This revised score was validated against clinical expert assessment in the primary evaluation as well as in estimations of therapeutic efficacy and in pre- and post-surgical assessments (Table 4).

**Table 4.** The St. Mark's (Vaizey) score

Type of incontinence	Frequency				
	Never	Rarely	Sometimes	Usually	Always
Solid	0	1	2	3	4
Liquid	0	1	2	3	4
Gas	0	1	2	3	4
Lifestyle alteration	0	1	2	3	4
				No	Yes
Need to wear a pad or plug				0	2
Taking constipating medicines				0	2
Lack of ability to defer defecation for 15 minutes				0	4

Never = no episodes in the past four weeks; Rarely = 1 episode in the past four weeks;

Sometimes = >1 episode in the past four weeks but <1 a week; Usually = 1 or more episodes a week but <1 a day; Always = 1 or more episodes a day.

Add one score from each row.

Minimum score is 0 = perfect continence; maximum score is 24 = totally incontinent.

The Pescatori AI score (Table 5) is a grading system widely used throughout Italy and also combines both degree of incontinence (flatus–mucus/liquid stool/solid stool) with frequency. Incontinence ratings of A, B and C indicate AI for flatus/mucus, liquid stool, and solid stool, respectively; frequency scores of 1, 2 and 3 indicate occasional, weekly, and daily AI. A score of zero is given for normal continence. The combined score is the sum of the degree and the frequency (e.g. A3 = 1 + 3 = 4; C2 = 3 + 2 = 5). The minimum score is 0 and the maximum score is C3 (= 6).

**Table 5.** The Pescatori incontinence score

	Degree	Frequency	
A	Incontinence for flatus/mucous	Less than once a week	1
		At least once a week	2
		Every day	3
B	Incontinence for liquid stool	Less than once a week	1
		At least once a week	2
		Every day	3
C	Incontinence for solid stool	Less than once a week	1
		At least once a week	2
		Every day	3

AI score = AI degree (A = 1, B = 2 or C = 3) + AI frequency.

## DISCUSSION

There is extensive evidence to support the impact of AI on patient-reported standardized quality of life and many aspects of healthy existence [40, 41], where the under-reporting of symptoms (and their specificity towards quality of life impairment) would suggest an even greater national annual economic cost of conservative (i.e. non-surgical) care of these patients than is currently recognized [42]. These analyses must consider the inherent additional costs of anti-diarrheal drugs, healthcare visits, intermittent hospitalizations and patients' payments for protective materials and pads. The additive costs of surgical therapies (given the marked shift in and expense of newer treatments such as sacral neuromodulation) are significant and are impacted by their long-term success rates, the economic impact of procedure-related complications (which are considerable with some of the newer therapies) and the incidence of corrective surgical procedures [43–45].

The risk factors for AI have been well established, in as much as the vast majority of cases develop because of a specific attributable insult: most notably obstetric trauma, anal surgery, neurological disease and after other pelvic surgery or sphincter irradiation. The incidence of occult anal sphincter injury is compounded by its poor correlation, even in the immediate post-partum period, with specific continence disturbance and it is likely that the problem is partly underestimated for this reason, given that even uneventful deliveries may have a much higher incidence of immediate continence disturbance as may minor anal surgical procedures. The documented risks for the subsequent development of AI include vacuum or forceps assisted vaginal delivery [46], inherent sphincter injury (in both primipara and multipara) [47], and pudendal and pelvic neuropathy [48, 49]. The other principal obstetric risk factors include the requirement for an episiotomy, a midline episiotomy, postpartum perineal sepsis, a large birth weight, cephalopelvic disproportion and a prolonged second stage of labor [50]. Further, these disorders, although obviated by a Caesarean section after a failed trial of labor, are not eliminated if there is an initial trial of vaginal labor [51].

The objective assessment of symptoms attributable to AI and their impact on health remain to be fully standardized in the context of an intention-to-treat basis. From the surgeon's point of view, objective data, such as the type of incontinence and the frequency of incontinent episodes, seem to be the most relevant aspects from which a surgical or non-surgical treatment option is proposed and evaluated. However, from the patient's perspective (which, in fact, should be more important and relevant in our evaluation), information related to the effect of hygiene, impact of continence on social activities and normal daily activities—as well as the occurrence and timing of episodes of leakage—are often the most worrisome specific personal issues. These systems are not perfect and do not generally incorporate psychometric evaluations and their interaction with other symptoms. More recently, attempts have been made to incorporate sexual dysfunction, urinary obstruction, fecal incontinence, obstructed defecation scores and urinary incontinence scoring in a dynamic map in which treatments affect map symmetry [52]. This so-called TAPE (three axial, perineal evaluation) score, designed by Altomare *et al.*, proposes a hexagonal schematic with positive and negative parameters for urinary, sexual and fecal function, in which the effect of surgical intervention changes the symmetry and area of the polygon for a rapid visual quantitative and qualitative determination of the effect of therapy on specific pelvic floor dysfunctions in the three main pelvic floor compartments.

**Conflict of interest:** none declared.
